# Case report: Remarkable response to sintilimab, lenvatinib, and nab-paclitaxel in postoperative metastatic chemotherapy-resistant combined hepatocellular-cholangiocarcinoma

**DOI:** 10.3389/fphar.2023.1190967

**Published:** 2023-10-13

**Authors:** Nan Zhou, Chuan-Fen Lei, Si-Rui Tan, Qi-Yue Huang, Shun-Yu Zhang, Zheng-Xin Liang, Hong-Feng Gou

**Affiliations:** ^1^ Department of Medical Oncology, Cancer Center, West China Hospital, Sichuan University, Chengdu, Sichuan, China; ^2^ Gastric Cancer Center, West China Hospital, Sichuan University, Chengdu, China; ^3^ Department of Pathology, West China Hospital, Sichuan University, Chengdu, China; ^4^ Department of Urology, The Second Hospital of Tianjin Medical University, Tianjin, China

**Keywords:** combined hepatocellular-cholangiocarcinoma (cHCC-CCA), sintilimab, lenvatinib, nabpaclitaxel, second-line treatment

## Abstract

**Background:** Combined hepatocellular-cholangiocarcinoma (cHCC-CCA) is a highly aggressive malignancy with a poor prognosis. However, there are no consensus treatment guidelines, and decisions are usually extrapolated from intrahepatic cholangiocarcinoma (ICC) or hepatocellular carcinoma (HCC). Given that cHCC-CCA owns the unequivocal presence of both hepatocytic and cholangiocytic differentiation, a combination regimen of anti-PD1 antibody, multikinase inhibitor, and chemotherapy targeting against both components might be an optimal choice.

**Case presentation:** We present the case of a patient with postoperative metastatic chemotherapy-resistant cHCC-CCA who exhibited a durable response and reasonable tolerability to a combination therapy consisting of the anti-PD1 antibody sintilimab, multikinase inhibitor lenvatinib, and nab-paclitaxel, despite having a low tumor mutational burden (TMB-L), microsatellite stability (MSS), and negative programmed cell death 1 ligand 1 (PD-L1).

**Conclusion:** The combination regimen of immune checkpoint inhibitor sintilimab, multikinase inhibitor lenvatinib, and chemotherapy with nab-paclitaxel, which targets both the HCC and ICC components, may represent a promising treatment option for patients with cHCC-CCA. Further research is warranted to validate these findings in larger patient cohorts.

## Background

Combined hepatocellular-cholangiocarcinoma (cHCC-CCA) is characterized by the coexistence of biliary and hepatocellular differentiation (Nagtegaal et al., 2020). The incidence of cHCC-CCA among the other primary liver cancers varies from 0.4% to 14.2% (Seehawer et al., 2019). The clinical outcome is rather poor due to tumor aggressiveness, higher risk of recurrence, and extra-hepatic involvement. Unfortunately, there are no established treatment guidelines for cHCC-CCA, and therapeutic decisions are often extrapolated from studies involving hepatocellular carcinoma (HCC) or intrahepatic cholangiocarcinoma (ICC). Despite efforts to adapt treatments from HCC or ICC, the clinical outcomes for cHCC-CCA remain suboptimal. In a multicenter retrospective study, the median overall survival (mOS) for patients treated with gemcitabine plus cisplatin, fluorouracil plus cisplatin, or sorafenib monotherapy in the first-line setting were only 11.9, 10.2, and 3.5 months, respectively (Kobayashi et al., 2018). Moreover, limited data suggest that second-line treatment outcomes are even less satisfactory. These results underscore the urgent need for more effective combination therapies tailored to the unique characteristics of cHCC-CCA.

The World Health Organization (WHO) classification defines cHCC-CCA as a primary liver carcinoma with the unequivocal presence of both hepatocytic and cholangiocytic differentiation within the same tumor on routine histopathology with H&E staining. While it is generally accepted that managing cHCC-CCA based on ICC principles may yield better results than approaches derived from HCC (Trikalinos et al., 2018), one study revealed that the patterns of recurrence or metastasis were similar in cHCC-CCA and HCC (Yen et al., 2021). Additionally, research has shown that cHCC-CCA shares a common cellular origin with HCC and genetically resembles HCC (Joseph et al., 2019). Given these shared characteristics with both HCC and ICC, a combination regimen targeting both components of cHCC-CCA may represent the optimal therapeutic approach.

In recent years, great progress has been made in ICC and HCC. For HCC, tyrosine kinase inhibitors (TKIs) sorafenib or lenvatinib monotherapy (Kudo et al., 2018) or a combination regimen of immune checkpoint inhibitors (ICIs) atezolizumab or sintilimab plus bevacizumab or bevacizumab biosimilar were approved as the first-line treatments for advanced HCC (Finn et al., 2020; Ren et al., 2021). For advanced biliary tract cancers (BTC), chemotherapy with or without ICI is the standard first-line treatment (Oh et al., 2022a). Nanoparticle albumin-bound (nab)-paclitaxel has demonstrated clinical benefits in both biliary tract cancer (BTC) and HCC (Zhou et al., 2011; Shroff et al., 2019; Talwar et al., 2020; Cheon et al., 2021). A phase 2 clinical trial has shown that treatment with nab-paclitaxel and gemcitabine-cisplatin (GC) had longer PFS and OS than GC alone (Shroff et al., 2019). The mechanisms underlying the antitumor effects of nab-paclitaxel include the promotion of tumor-associated antigen release, increased infiltration of tumor-infiltrating lymphocytes, and enhanced tumor cell permeability to granzyme (Weiss et al., 2017; Martin et al., 2020; Peng et al., 2022). Based on these encouraging results, combination therapy consisting of sintilimab, lenvatinib, and chemotherapy might be an optimal choice for cHCC-CCA.

Here, we present the first case of a patient with postoperative metastatic cHCC-CCA who achieved a partial response (PR) following second-line treatment of sintilimab, lenvatinib, and nab-paclitaxel.

## Case presentation

In June 2019, a 53-year-old male was incidentally found to have a liver-occupying lesion during a routine physical examination. Subsequent contrast-enhanced magnetic resonance imaging (MRI) identified a solitary lesion measuring 2.7 cm × 2.3 cm in the left medial segment of the liver. Liver function tests and tumor markers fell within the normal range. The patient had a 20-year history of hepatitis B but had not received antiviral treatment. Additionally, he had a history of occasional alcohol consumption and smoking. Notably, there was a family history of liver cancer in his mother.

**FIGURE 1:**
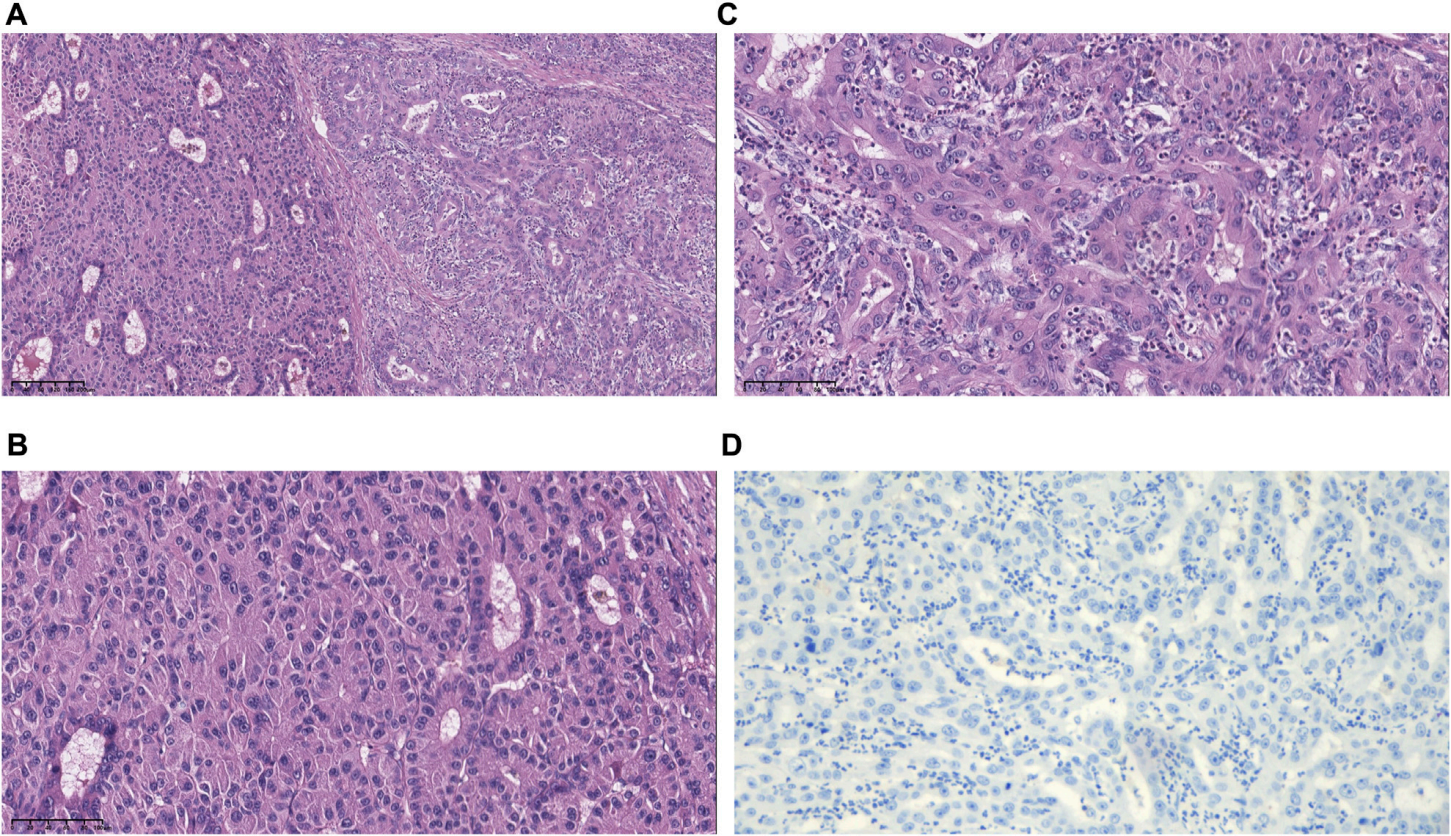
Morphological and immunohistochemical features of specimens. **(A)** Hematoxylin and eosin (H&E)-stained image showing hepatocellular and biliary epithelial differentiation within a single tumor nodule. **(B)** Higher magnification of the hepatocellular carcinoma (HCC) region shows an unequivocal hepatocellular area composed of large tumor cells with abundant eosinophilic cytoplasm. **(C)** Higher magnification of the intrahepatic cholangiocarcinoma (ICC) region shows an unequivocal adenocarcinoma area demonstrating clear gland formation. **(D)** The tumor was PD-L1 negative with CPS <1, TPS <1 (magnification, ×10).

**FIGURE 2:**
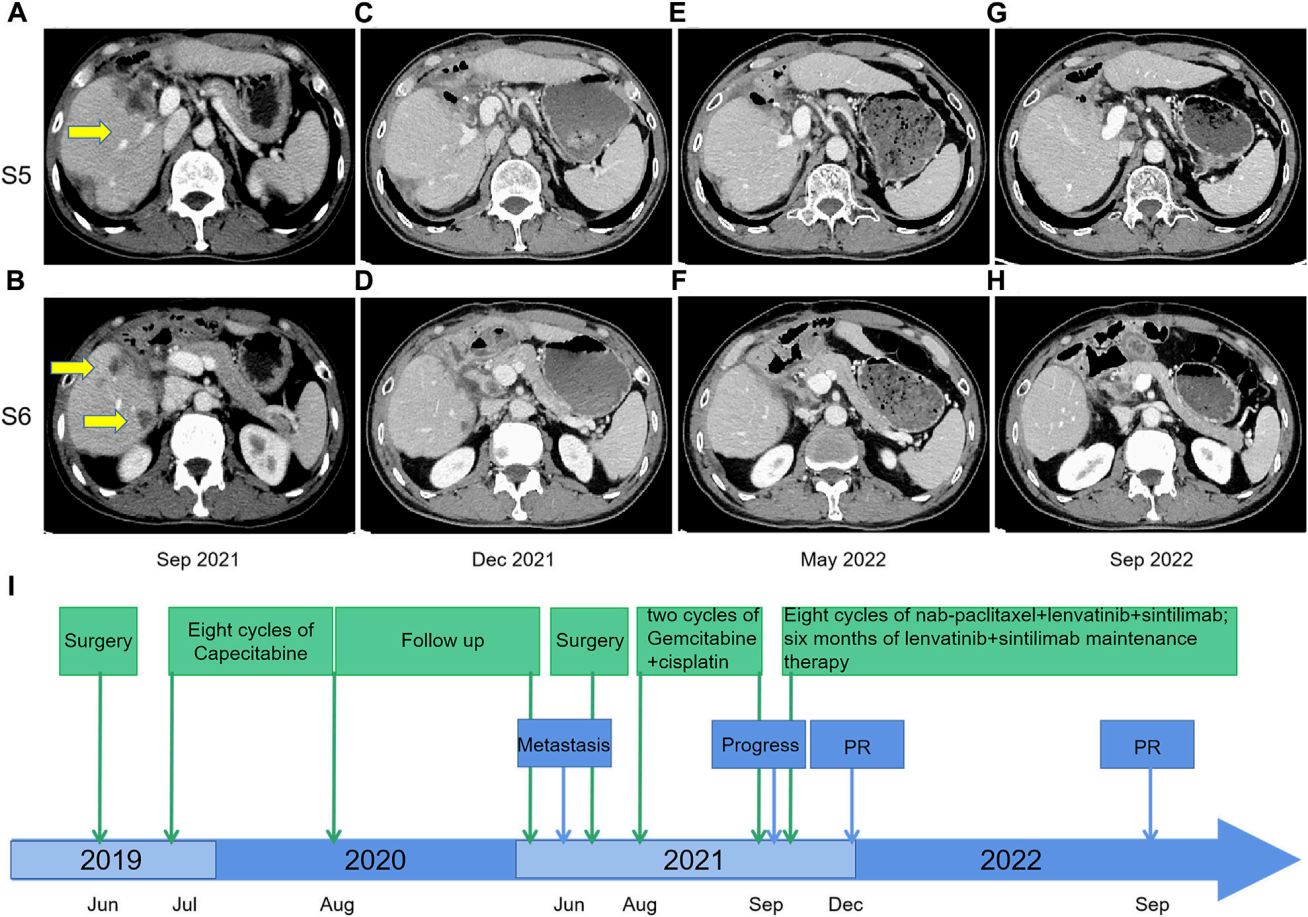
Contrast-enhanced CT scans before and after nab-paclitaxel in combination with lenvatinib and sintilimab treatment; **(A,B)** September 2021: tumor recurrence and multiple metastases were shown in the liver after two courses of gemcitabine-cisplatin chemotherapy; **(C,D)** December 2021: response to nab-paclitaxel, lenvatinib, and sintilimab therapy, with size reduction of defined target lesions greater than 30% which corresponds to partial response; **(E,F)** May 2022: the partial response remained after eight courses of triple therapy; **(G,H)** September 2022: The CT scan indicated continuing tumor control with maintenance therapy of lenvatinib plus sintilimab from May 2022 to September 2022; **(I)** Timeline from June 2019–December 2022.

On 17 June 2019, the patient underwent hepatectomy and cholecystectomy. Postoperative histological examination confirmed the diagnosis of combined hepatocellular-cholangiocarcinoma (cHCC-CCA) with cirrhosis (Figures 1A–C). Following surgery, the patient received adjuvant chemotherapy with capecitabine for 8 cycles, after which regular follow-up was initiated. However, in June 2021, abdominal computed tomography (CT) demonstrated metastatic liver masses in segments IV and VII, measuring 1.7 cm × 1.5 cm and 3.4 cm × 3.2 cm, respectively. Subsequently, right and middle hepatectomies were performed, achieving negative surgical margins. The patient then received GC chemotherapy as first-line treatment. Regrettably, after just two cycles, a CT scan showed tumor recurrence and multiple intrahepatic metastases (Figures 2A, B). The largest metastasis was located at the surgical margin, measuring approximately 3.0 cm × 2.5 cm. Genomic analysis revealed that the tumor with low tumor mutational burden (TMB-L), microsatellite stability (MSS), and negative PD-L1 expression (Figure 1D). Additionally, several gene mutations were identified, including PTEN, TERT, GNAQ, FAT2, ROS1, CTNNB1, and ERBB4.

The second-line treatment of the patient was discussed by the multidisciplinary team. Considering the features of cHCC-CCA, we initiated a novel treatment regimen comprising nab-paclitaxel (125 mg/m2, on days 1 and 8), lenvatinib (8 mg/day), and sintilimab (200 mg) every 21 days. Remarkably, after just two treatment cycles, a noticeable reduction in tumor size was observed, resulting in a partial response (PR) (Figures 2C, D). A total of eight cycles of the triple therapy were administered (Figures 2E, F), followed by 6 months of maintenance treatment with lenvatinib and sintilimab. The CT scan in September 2022 indicated continued tumor control (Figures 2G, H). Treatment-related adverse events were limited to grade 2 neutropenia, grade 1 leukopenia, and grade 1 peripheral neuropathy, with no events necessitating treatment discontinuation. Treatment-related adverse events were limited to grade 2 neutropenia, grade 1 leukopenia, and grade 1 peripheral neuropathy, with no events necessitating treatment discontinuation. The timeline is illustrated in Figure 2I.

## Discussion

Combined hepatocellular-cholangiocarcinoma is characterized by aggressive behavior and a dismal prognosis. However, no therapy has been established for patients with cHCC-CCA. Sorafenib, the first-line treatment for HCC, and GC, the standard of care for ICC, do not seem to have significant antitumor efficacy. The objective response rate (ORR) was as low as 28.6% and the median progress-free survival (PFS) was 9.0 months with gemcitabine plus platinum-based regimen (Salimon et al., 2018). Besides, the median overall survival (mOS) of sorafenib monotherapy groups was merely 3.5 months (Kobayashi et al., 2018). Second-line therapies yielded less desirable outcomes, with an analysis of 44 patients indicating a PR rate of only 2.3% and median PFS and OS of 2.2 months and 9.2 months, respectively (Kim et al., 2021). Our case report demonstrates a patient with postoperative metastatic cHCC-CCA who achieved a partial response (PR) after two cycles of treatment comprising sintilimab, lenvatinib, and nab-paclitaxel. This suggests that combination therapies targeting both the HCC and ICC components may hold promise for cHCC-CCA. This strategy warrants further exploration, especially in the context of front-line treatment.

The response to immunotherapy is sometimes associated with TMB, PD-L1 expression, MMR, or MSI-H. Some malignancies, including biliary tract cancer (BTC) and HCC, have shown that patients with high TMB (TMB-H) or those with deficient mismatch repair/microsatellite instability-high (dMMR/MSI-H) status can benefit from immunotherapy (Czink et al., 2017; Marabelle et al., 2020; Zhang et al., 2021). The correlation between PD-L1 expression and the efficacy of ICIs remains uncertain. The response rate to immunotherapy was found to range from 0% to 17% in PD-L1- negative BTC patients (Patel and Kurzrock, 2015). Besides, the absence of a relationship between PD L1 status and clinical activity with chemoimmunotherapy was revealed in HCC and ICC (Lee et al., 2020; Oh et al., 2022b). Our patient demonstrated survival benefits despite having a TMB-L, PD-L1-negative, and MSS status.

Several factors may contribute to this remarkable response, including potential variations in PD-L1 assessment techniques (Paavilainen et al., 2010; Rimm et al., 2017), the dynamic nature of PD-L1 expression (Rimm et al., 2017; Wang et al., 2023), and the potential additive or synergistic effects of triple therapy. Combination treatment of chemotherapy, immunotherapy, and targeted therapy has provided superior efficacy with reasonable tolerability in several types of cancers (Reck et al., 2019; Zhou et al., 2020; Dong et al., 2022; Lu et al., 2022). In our case, therapy of sintilimab plus lenvatinib plus nab-paclitaxel was efficacious and well tolerated in patient with advanced cHCC-CCA. In addition to the known antiangiogenic effects, the inhibition of vascular endothelial growth factor (VEGF) such as lenvatinib has immunomodulatory effects (Voron et al., 2015). The efficacy of sintilimab may be enhanced through the addition of lenvatinib to reverse VEGF-mediated immunosuppression. Additionally, following treatment with chemotherapy, dying cancer cells can stimulate dendritic cells, which enhances antigen presentation and facilitates the priming of CD8^+^ tumor-specific T cells (Apetoh et al., 2007; Ghiringhelli et al., 2009). The addition of sintilimab to lenvatinib and chemotherapy, both of which have immunomodulatory effects that may augment the efficacy of sintilimab (Chen and Mellman, 2013; Hegde et al., 2018). In patients with advanced BTC, the combination of chemotherapy, sintilimab, and lenvatinib led to significant benefits, with an ORR of 45.5% and a disease control rate (DCR) of 86.4% (Dong et al., 2022). Based on these encouraging discoveries, triple modalities might be a logical next step for cHCC-CCA.

## Conclusion

In summary, a combination regimen incorporating sintilimab, lenvatinib, and nab-paclitaxel, addressing both HCC and ICC components, offers a potential treatment option for cHCC-CCA. This case provides a rationale for further investigation in clinical trials.

## Data Availability

The original contributions presented in the study are included in the article/Supplementary Material, further inquiries can be directed to the corresponding author.
